# Lenvatinib combined with anti-PD-1 antibodies plus locoregional treatment for initial unresectable hepatocellular carcinoma with portal vein tumor thrombosis: a multicenter real-world study

**DOI:** 10.1186/s12885-025-14543-9

**Published:** 2025-07-10

**Authors:** Qi-Yu Chi, Quan-Yuan Liu, Shuang-Jia Wang, Ye-Dong Liu, Xiao-Di Gao, Kong-Ying Lin, Lan-Fang Yang, Zi-Jian Liu, Min-Hui Chi, Shan-Geng Weng, Yong-Yi Zeng, Zhi-Bo Zhang

**Affiliations:** 1 Department of Hepatopancreatobiliary Surgery, The First Affiliated Hospital of Fujian Medical University, Fuzhou, China; 2 Department of Hepatopancreatobiliary Surgery, National Regional Medical Center, Binhai Campus of the First Affiliated Hospital, Fujian Medical University, Fuzhou, China; 3 Department of Hepatobiliary Surgery, The Zhangzhou Affiliated Hospital of Fujian Medical University, Zhangzhou, China; 4 Department of Hepato-Biliary-Pancreatic and Vascular Surgery, The First Affiliated Hospital of Xiamen University, Xiamen, China; 5 Department of Hepatopancreatobiliary Surgery, Mengchao Hepatobiliary Hospital of Fujian Medical University, Fuzhou, China

**Keywords:** Unresectable hepatocellular carcinoma, Portal vein tumor thrombosis, Systemic therapy, Locoregional therapy, Surgery

## Abstract

**Background:**

Unresectable hepatocellular carcinoma (uHCC) with portal vein tumor thrombosis (PVTT) has poor prognoses. This study evaluated the efficacy and safety of lenvatinib (LEN) combined with anti-PD-1 antibodies (PD-1) and locoregional therapy (LRT) in uHCC patients with PVTT.

**Methods:**

Consecutive uHCC patients with PVTT who received LEN, PD-1, and LRT (LPLRT), such as transcatheter arterial chemoembolization (TACE), hepatic arterial infusion chemotherapy (HAIC), or TACE-HAIC, were analyzed. Objective response rate (ORR), disease control rate (DCR), overall survival (OS), progression-free survival (PFS), and treatment-related adverse events (TRAEs) were assessed. Subgroup analysis and multivariate Cox regression analysis was performed to identify independent risk factors for OS and PFS.

**Results:**

Between January 2019 and December 2021, 74 uHCC patients with PVTT at four tertiary hospitals were enrolled. Of these, 38 were treated with LEN, PD-1, and TACE (LPT), 12 with LEN, PD-1, and HAIC (LPH), and 24 with LEN, PD-1, and TACE-HAIC (LPTH). According to the modified Response Evaluation Criteria in Solid Tumors, the ORR and DCR were 62.1% and 85.1%, respectively. The median OS was 23.3 months (95% CI, 18.9–27.7 months), and the median PFS was 13.2 months (95% CI, 8.8–17.6 months). Subgroup analyses revealed no significant differences in ORR, DCR, OS or PFS among the LPT, LPH, and LPTH groups. No grade 5 TRAEs occurred. Twenty-nine patients underwent salvage surgery. Significant differences in OS and PFS rates were observed between the resection and non-resection groups (*p* < 0.001 for both). Multivariate analysis showed that surgical resection was an independent prognostic factor for OS and PFS.

**Conclusion:**

LPLRT therapy offers a promising treatment for uHCC patients with PVTT, demonstrating high tumor response and conversion rates, prolonged survival, and manageable safety. Notably, for patients who remain eligible for surgery following LPLRT, salvage surgery is a safe, effective, and potentially prognostic treatment option.

**Supplementary Information:**

The online version contains supplementary material available at 10.1186/s12885-025-14543-9.

## Introduction

Hepatocellular carcinoma (HCC) remains a significant global health challenge, being the sixth most common cancer and the third leading cause of cancer-related death [1]. Most HCC patients are diagnosed at an advanced stage or have concomitant liver insufficiency, thus losing the opportunity for radical surgery [2, 3]. Given HCC’s propensity to invade the portal vein and its branches, a substantial proportion of these advanced patients develop portal vein tumor thrombosis (PVTT), further complicating treatment efforts [4, 5]. Due to the disease’s aggressive nature and the limited efficacy of conventional therapies, prognosis remains poor [6], underscoring the urgent need for new treatment strategies to improve clinical outcomes.

Conversion therapy aims to downstage initially unresectable tumors, offering patients the chance for radical resection. Although traditional locoregional therapies (LRT) have been employed for many years [7–9], their conversion rates remain limited [8, 10]. Recent advances highlight the potential of combining systemic therapy with LRT to achieve better tumor control and anti-PD-1 antibodies improve survival rates [11–14]. Potent tyrosine kinase inhibitors like lenvatinib (LEN) and anti-PD-1 antibodies (PD-1) have shown significant promise in treating advanced HCC, with studies demonstrating that combining these systemic therapies with LRT such as transcatheter arterial chemoembolization (TACE) or hepatic arterial infusion chemotherapy (HAIC) can improve response rates and overall survival (OS) [15–18]. However, the presence of PVTT, particularly Vp2-Vp4 types, complicates treatment due to the high risk of vascular invasion and rapid disease progression.

Currently, the Barcelona Clinic Liver Cancer (BCLC) staging system recommends systemic treatment for HCC patients with PVTT using targeted therapy and immunotherapy [19–21]. According to the recommendations of the Chinese Liver Cancer (CNLC) guidelines, for HCC patients with PVTT, except for a very small number of strictly screened patients who meet specific conditions, liver resection can be considered safely [22]. In most cases, the guidelines emphasize the use of comprehensive therapy that combines LRT with systemic therapy in order to significantly improve the patient’s treatment effect and improve the prognosis [12]. This combination therapy can modulate the tumor microenvironment, enhance the efficacy of immune checkpoint inhibitors, and improve clinical outcomes [14]. Recent clinical studies have shown that the highest objective response rate (ORR) of LEN combined with PD-1 and TACE or HAIC in treating advanced HCC patients with PVTT were 68.9% and 82.4%, respectively, demonstrating significant efficacy [23]. Another phase II study explored the combination of LEN and toripalimab with HAIC for the treatment of advanced HCC. Among the 36 patients included, 86.1% had PVTT, and the ORR with combined treatment was as high as 63.9%, indicating promising results [24]. Although these findings are encouraging, there is a lack of real-world data on the efficacy and safety of this treatment for patients with unresectable HCC (uHCC) with PVTT.

Therefore, this multicenter real-world study aims to evaluate the clinical benefits of LEN combined with PD-1s plus LRT such as TACE, HAIC, or a combination of TACE and HAIC (TACE-HAIC) in patients with initially uHCC with PVTT. Our goal was to assess the efficacy and safety of this combination therapy while also comparing these parameters across different subgroups. This study aims to provide valuable insights into the potential of this treatment regimen as a viable option for this challenging patient population.

## Patients and methods

### Patients

A retrospective analysis was conducted on patients with unresectable HCC (uHCC) with PVTT who received LEN, PD-1, and LRT (LPLRT) between January 2019 and December 2021 at four large tertiary hospitals in China: First Affiliated Hospital of Fujian Medical University, Zhangzhou Affiliated Hospital of Fujian Medical University, Mengchao Hepatobiliary Hospital of Fujian Medical University, and First Affiliated Hospital of Xiamen University. The diagnosis of HCC conformed to both the American Association for the Study of Liver Diseases practice guideline and the Chinese Guidelines for the Diagnosis and Treatment of Primary Liver Cancer [25]. UHCC was defined according to the Liver Cancer Study Group of Japan (LCSGJ) criteria [26], which includes extensive bilobar involvement of the liver due to a large solitary tumor or multiple tumors, or invasion of major vessels, including the main trunk of the portal vein (Vp4) and the inferior vena cava (Vv3). The diagnosis of PVTT was based on typical radiological features observed in imaging studies or confirmed through histological examinations [25].

The inclusion criteria included the following: (1) uHCC with Vp2-Vp4; (2) initial treatment with LEN, PD-1, and LRT (TACE, HAIC, or TACE combined with HAIC); (3) age between 18 and 75 years; (4) Child-Pugh class A or B (≤ 7 points); (5) at least one measurable target lesion according to modified Response Evaluation Criteria in Solid Tumors (mRECIST) criteria; and (6) Eastern Cooperative Oncology Group (ECOG) performance status score of 0–1. The exclusion criteria included the following: (1) prior treatment with systemic therapy or LRT; (2) a history of resection of liver cancer; (3) severe hepatic, renal, or cardiopulmonary insufficiency; (4) a history of other cancers; (5) incomplete data; (6) inability to tolerate treatment due to personal reasons.

This study complied with the ethical principles of the Declaration of Helsinki and was approved by the Ethics Review for Medical Research and Clinical Technology Application, and Research Ethics Committee of First Affiliated Hospital of Fujian University (Approval No. 2024 − 587). Written informed consent was obtained from each patient before they received targeted therapy combined with immunotherapy and LRT, surgery, and before the collection of their clinicopathological data.

### Conversion therapy

All patients received LEN orally once daily (8 mg for those with a body weight < 60 kg and 12 mg for those with a body weight ≥ 60 kg) and PD-1s (camrelizumab 200 mg, tislelizumab 200 mg, sintilimab 200 mg, or toripalimab 240 mg) intravenously every 3 weeks. The selection of PD-1s was based on the latest efficacy and safety data, the patients’ economic status, and drug availability at each center. Concurrently, LRT was tailored according to the patient’s liver function, ECOG-PS, tumor burden, presence of capsule, presence of arteriovenous shunts, and portal vein thrombosis. LEN was discontinued for at least 3 days before and after each LRT.

TACE was performed under local anesthesia. Selective hepatic angiography was utilized to identify the tumor-feeding vessels. An emulsion of iodized oil, platinum-based drugs, and/or pirarubicin was then injected into the identified vessels through a microcatheter. This was followed by embolization with gelatin sponge particles until complete stasis of arterial blood flow was achieved. The TACE treatment was generally repeated every 4–6 weeks.

HAIC was also performed under local anesthesia, using the modified Seldinger technique to insert a catheter through the femoral artery percutaneously, and retain the microcatheter in the tumor supply artery. If the intrahepatic tumor was supplied by a single left or right hepatic artery, the microcatheter was inserted into the corresponding target vessel. If the intrahepatic tumor was supplied by the left and right hepatic arteries, the microcatheter was inserted into the proper hepatic artery. To protect the normal gastrointestinal tract, if the gastroduodenal artery could not be avoided, the gastroduodenal artery was embolized with a coil. The chemotherapy perfusion dose of the FOLFOX regimen was: oxaliplatin 85mg/m^2^ arterial infusion for 2 hours, calcium folinate 400mg/m^2^ intravenous infusion for 1 h, and then combined with 5-fluorouracil 400mg/m^2^ arterial infusion for 1 h, and 5-fluorouracil 2400mg/m^2^ arterial infusion for 23 h, repeated once every 3 to 4 weeks. The drug dose could be adjusted according to chemotherapy tolerance. If the tumor was significantly reduced after HAIC treatment, the chemotherapy drug dose could be appropriately reduced.

TACE-HAIC was categorized into simultaneous and non-simultaneous regimens. For patients who received TACE in addition to LEN and PD-1, if the therapeutic effect was deemed unsatisfactory upon review with upper abdominal enhanced CT and/or MRI, TACE in the LPLRT was sometimes replaced with HAIC, constituting the non-simultaneous approach. Currently, most TACE combined with HAIC procedures are performed simultaneously. The chemotherapy embolization method for the TACE-HAIC therapy was consistent with the aforementioned technique. Afterward, a microcatheter was placed and secured in the tumor-feeding artery, and chemotherapy infusion based on the FOLFOX regimen was administered. The recommended dose was the same as in the HAIC procedure. TACE-HAIC treatment was generally repeated every 4 to 6 weeks.

### Patient management

All patients with active HBV infection received oral entecavir or tenofovir as antiviral treatment, which was continued throughout the anticancer therapy. For patients with resectable HCC (rHCC) who were scheduled for surgery, LEN was discontinued at least 1 week before, and PD-1 inhibitors were discontinued at least 2 weeks before the procedure. It is recommended to resume LEN and PD-1 as adjuvant therapy within 4 weeks post-surgery, with adjuvant therapy lasting 6 to 12 months. Follow-up was conducted every 3 months during adjuvant therapy. If two consecutive imaging examinations showed no tumor metastasis or recurrence and tumor markers remained normal for 3 months without an upward trend, drug discontinuation could be considered.

The criteria for rHCC followed our previous work [14]. Patients considered to have achieved successful conversion were evaluated through multidisciplinary team (MDT) discussions and underwent surgical resection performed by the lead investigator at each participating center. The eligibility criteria for rHCC included the following: (1) sufficient future liver remnant, defined as ≥ 40% of the total liver volume in patients with cirrhosis and ≥ 30% in non-cirrhotic patients; (2) the potential to achieve an R0 resection with adequate margins; (3) preserved hepatic function, meeting Child–Pugh class A; (4) absence of severe adverse events during conversion therapy; and (5) no contraindications to major hepatectomy. Patients who did not meet surgical criteria continued to receive LPLRT therapy until disease progression, intolerable toxicity, refusal of treatment, or other situations deemed necessary for treatment discontinuation by the researchers.

### Clinical evaluations

Before treatment, all patients underwent comprehensive evaluations, including detailed medical histories, physical examinations, and blood tests (covering routine blood work, liver and kidney function, coagulation, thyroid and cardiac function, hepatitis B surface antigen [HBsAg], HBV-DNA, serum alpha-fetoprotein [AFP], and vitamin K deficiency-induced protein-II [PIVKA-II]). Urine analysis and baseline radiological examinations (liver-enhanced CT and/or MRI, and chest CT) were also conducted. During treatment, follow-up visits occurred every three weeks, incorporating history collection, physical examinations, and laboratory tests. Radiological evaluations (CT/MRI) were performed every two months (6–8 weeks) to assess intrahepatic tumors and lung conditions. Tumor assessment adhered to the HCC-specific mRECIST criteria. Throughout the treatment period, treatment-related adverse events (TRAEs) were documented and assessed based on the Common Terminology Criteria for Adverse Events version 5.0. Pathologic complete response (pCR) was defined as the complete absence of viable tumor cells in the resected specimen. Major pathologic response (MPR) was defined as having ≤ 10% of viable tumor cells in the resected specimen. After discontinuing treatment, all patients were monitored for survival with follow-up assessments every three months during the first and second years, and every six months thereafter.

### Statistical analysis

The statistical significance of differences in categorical variables was assessed using either the chi-square test or Fisher’s exact test. Continuous variables were expressed as median (interquartile range [IQR]) or mean ± SD and were compared using the t-test or one-way analysis of variance. Time to response (TTR) was defined as the interval from the start of treatment to the achievement of partial response (PR) or complete response (CR). Overall survival (OS) was defined as the interval between the initiation of combination therapy and the date of death. Time to recurrence was defined as the interval between the date of surgery and the date of tumor recurrence diagnosis. Survival at each time point was estimated using the Kaplan-Meier method. OS and progression-free survival (PFS) were compared between groups using the log-rank test. Multivariate analysis was performed using the Cox regression model. P values less than 0.05 were considered statistically significant. All statistical analyses were performed using the Statistical Package for the Social Sciences (SPSS) software (version 26, SPSS, Inc., Chicago, IL, USA).

## Results

### Patient characteristics at baseline

**Fig. 1:**
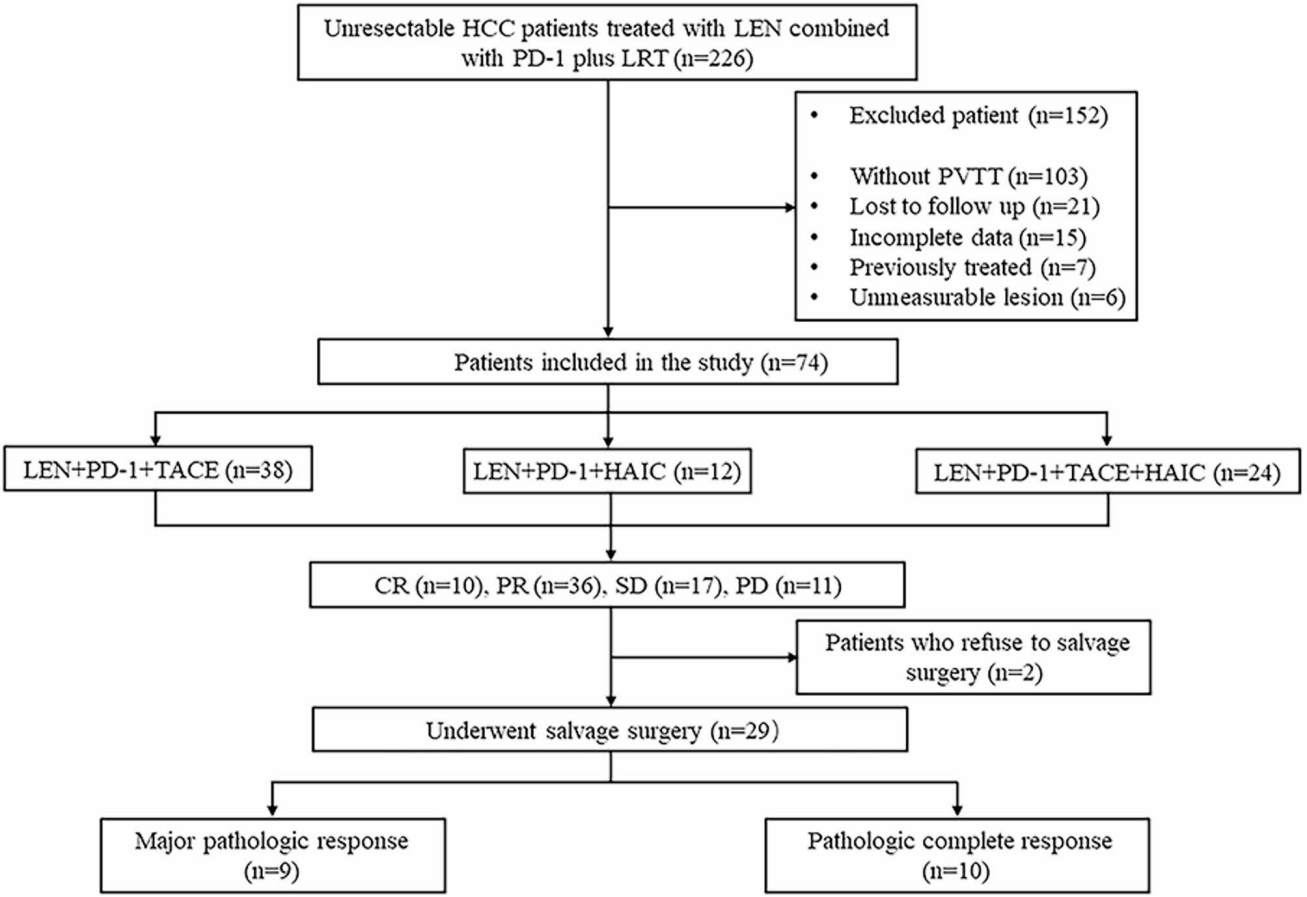
Flow chart of study design. HCC, hepatocellular carcinoma; LEN, lenvatinib; PD-1, anti-PD-1 antibodies; LRT, locoregional therapy; PVTT, Portal Vein Tumor Thrombosis; TACE, transcatheter arterial chemoembolization; HAIC, hepatic arterial infusion chemotherapy; CR, complete response; PR, partial response; SD, stable disease; PD, progressive disease

Between January 2019 and December 2023, a total of 226 uHCC patients received LPLRT therapy at four tertiary hospitals in China. Out of these, 123 patients were diagnosed with PVTT. A total of 152 patients were excluded from the study. Consequently, 74 uHCC patients with PVTT who were initially treated by LPLRT therapy were analyzed (Fig. 1). Of these 74 patients, 38 were treated with LEN, PD-1, and TACE (LPT), 12 with LEN, PD-1, and HAIC (LPH), and 24 with LEN, PD-1, and TACE-HAIC (LPTH). Thirty-one patients (41.9%) achieved successful conversion to rHCC, of whom 2 refused salvage surgery, 1 continued to receive the LPT therapy, and 1 receive the LPTH therapy. A total of 29 (39.2%) patients underwent salvage surgery.

The Baseline and disease characteristics of the included patients are summarized in Table 1. Of the 74 patients, the median age was 54.5 years (IQR, 26–84 years), with 51 patients (31.1%) aged ≥ 60 years and 67 (90.5%) being male. Up to 61 patients (82.4%) were infected with hepatitis B virus, and 41 patients (55.4%) had comorbidities. Baseline AFP was ≥ 400 ng/mL in 44 patients (59.5%) and baseline PIVKA-II was ≥ 400 mAU/mL in 52 patients (70.3%). The mean maximum tumor diameter was 11.6 ± 3.5 cm, with 48 patients (64.9%) having a tumor diameter greater than 10 cm. Forty-two patients (56.8%) had multiple tumors, and 21 (28.4%) had extrahepatic metastases (EHM). All patients were categorized as BCLC stage C. In subgroup analyses, clinical and disease features were found to be comparable, except for the number of tumors (Table 1). The number of tumors in the LPH and LPTH groups was higher than that in the LPT group (Table S1).

**Table 1:** Patient demographics and disease characteristics

Characteristic		Total (*n* = 74)		LPT (*n* = 38)	LPH (*n* = 12)	LPTH (*n* = 24)	*P* value
Gender n (%)							1.000*
Male		67 (90.5)		34 (89.5)	11 (91.7)	22 (91.7)	
Female		7 (9.5)		4 (10.5)	1 (8.3)	2 (8.3)	
Age (years), median (IQR)		54.5(26–84)		57 (26–80)	53.5 (45–80)	51.5 (31–84)	0.486
Age, years, n (%)							0.546
<60		51 (68.9)		24 (63.2)	9 (75.0)	18 (75.0)	
≥ 60		23 (31.1)		14 (36.8)	3 (25.0)	6 (25.0)	
ECOG PS, n (%)							1.000*
0		65 (87.8)		33 (86.8)	11 (91.7)	21 (87.5)	
1		9 (12.2)		5 (15.2)	1 (8.3)	3 (12.5)	
Hepatitis B infection, n (%)							0.981
No		13 (17.6)		7 (18.4)	2 (16.7)	4 (16.7)	
Yes		61 (82.4)		31 (81.6)	10 (83.3)	20 (83.3)	
HBV-DNA copy, n (%)							0.950
< 1000 copy/mL		34 (45.9)		17 (44.7)	6 (50.0)	11 (45.8)	
≥ 1000 copy/mL		40 (54.1)		21 (55.3)	6 (50.0)	13 (54.2)	
Comorbidity, n (%)							0.252
No		33 (44.6)		17 (44.7)	3 (25.0)	13 (54.2)	
Yes		41 (55.4)		21 (55.3)	9 (75.0)	11 (45.8)	
Child Pugh, n (%)							0.488
A		52 (70.3)		29 (76.3)	8 (66.7)	15 (62.5)	
B	22 (29.7)		9 (23.7)		4 (33.3)	9 (37.5)	
Baseline AFP, n (%)							0.348
< 400 ng/mL		30 (40.5)		15 (39.5)	3 (25.0)	12 (50.0)	
≥400 ng/mL		44 (59.5)		23 (60.5)	9 (75.0)	12 (50.0)	
Baseline PIVKA-II, n (%)							0.184
< 400 mAU/mL		22 (29.7)		12 (31.6)	1 (8.3)	9 (37.5)	
≥400 mAU/mL		52 (70.3)		26 (68.4)	11 (91.7)	15 (62.5)	
Tumor number, n (%)							0.032
Solitary		32 (43.2)		22 (57.9)	3 (25.0)	7 (29.2)	
Multiple		42 (56.8)		16 (42.1)	9 (75.0)	17 (70.8)	
Maximum tumor size(cm), mean ± SD		11.6 ± 3.5		11.0 ± 3.6	12.0 ± 2.9	12.2 ± 3.5	0.385
Tumor size, cm, n (%)							0.385
< 10		26 (35.1)		16 (42.1)	4 (33.3)	6 (25.0)	
≥ 10		48 (64.9)		22 (57.9)	8 (66.7)	18 (75.0)	
EHM, n (%)							0.098
No		53 (71.6)		25 (65.8)	7 (58.3)	21 (87.5)	
Yes		21 (28.4)		13 (34.2)	5 (41.7)	3 (12.5)	
PVTT ^a^, n (%)							0.172*
Vp2		23 (31.1)		12 (31.6)	2 (16.7)	9 (37.5)	
Vp3		31 (41.9)		18 (47.4)	3 (25.0)	10 (26.3)	
Vp4		20 (27.0)		8 (21.0)	7 (58.3)	5 (13.2)	
HVTT ^a^, n (%)							0.326*
Vv0-1		55 (74.3)		27 (71.1)	8 (66.7)	20 (83.3)	
Vv2		12 (16.2)		8 (21.1)	3 (25.0)	1 (4.2)	
Vv3		7 (9.5)		3 (7.8)	1 (8.3)	3 (12.5)	
CNLC staging, n (%)							0.098
IIIa		53 (71.6)		25 (65.8)	7 (58.3)	21 (87.5)	
IIIb		21 (28.4)		13 (34.2)	5 (41.7)	3 (12.5)	

*Abbreviations*: *ECOG-PS* Eastern Cooperative Oncology Group performance status, *AFP* α-fetoprotein, *PIVKA-II*, protein induced by vitamin K absence-II, *EHM* Extrahepatic metastasis, *PVTT* Portal vein tumor thrombosis, *HVTT* Hepatic vein tumor thrombosis, *CNLC* China liver cancer staging, *LEN* Lenvatinib, *PD-1* Anti-PD-1 Antibodies, *HAIC* hepatic arterial infusion chemotherapy, *TACE* transcatheter arterial chemoembolization

^a^Portal vein invasion and Hepatic vein tumor thrombus are graded according to the Liver Cancer Study Group of Japan

*Fisher's exact test. LPT, LEN+PD-1+TACE; LPH, LEN+ PD-1+HAIC; LPTH, LEN+ PD-1+TACE+HAIC

### Conversion therapy

The most common PD-1s used in all patients were Camrelizumab (48, 64.9%), followed by Tislelizumab (16, 21.6%), Sintilimab (9, 12.2%), Toripalimab (1, 1.3%). Camrelizumab was the most common PD-1 used in each subgroup, and there was no statistically significant difference in the type of PD-1s used in subgroup analyses (Table S2). The median duration of LEN treatment was 2.2 months (IQR, 1.1-15.7months). The median cycle of PD-1 treatment was 3 (IQR, 1–22). The median number of TACE and HAIC treatments both were 2 (IQR,1–6). There was no difference in the median duration of LEN treatment and the median duration of PD-1 treatment in subgroup analyses.

### Efficacy

**Fig. 2:**
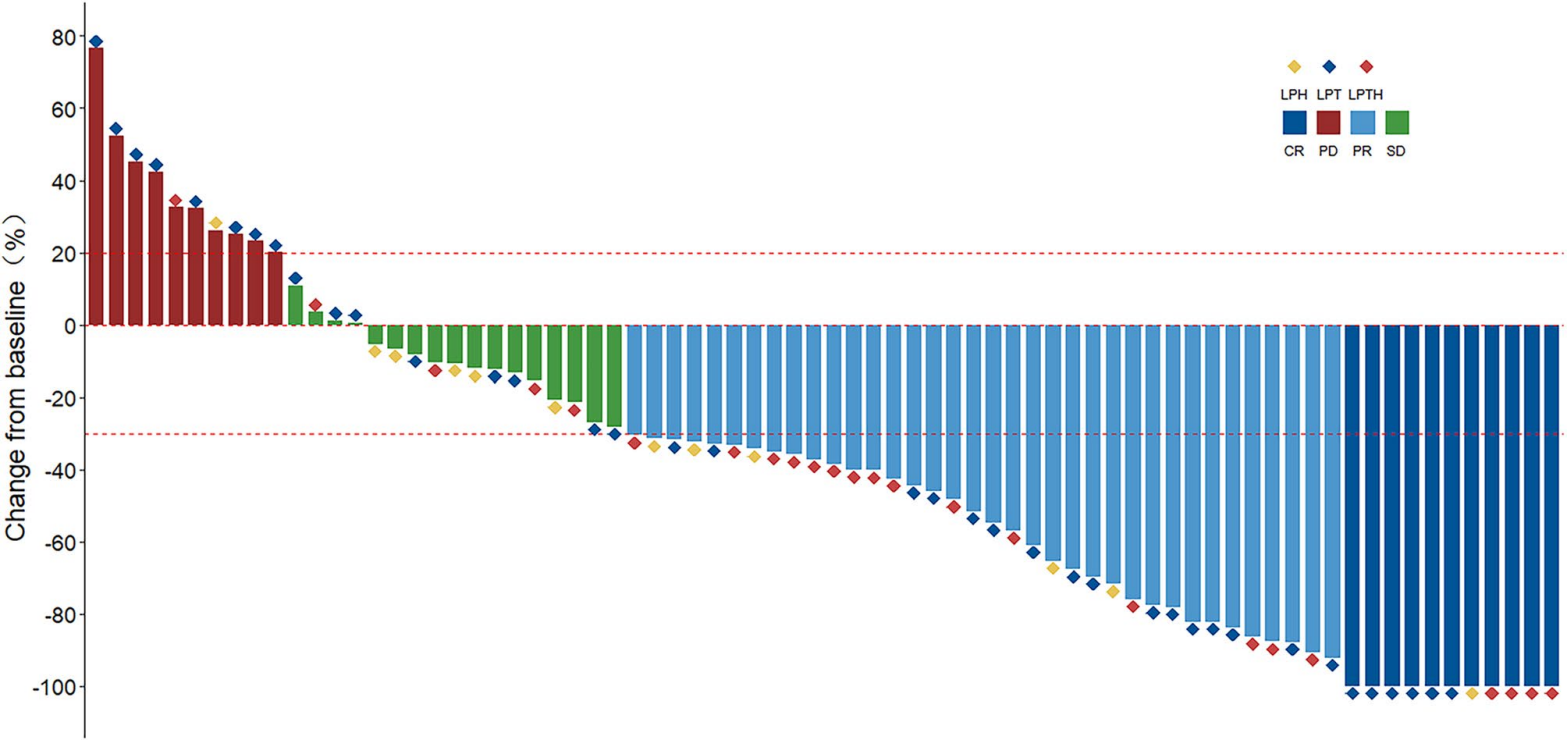
Waterfall plot of maximum tumor response to LPLRT therapy using the mRECIST criteria

As of data cutoff on March 1, 2024, the median follow-up was 16.1 months (IQR 1.6–43.8 months). The best responses are shown in Table 2. According to the mRECIST criteria, complete response (CR) was achieved in 10 patients (13.5%), partial response (PR) in 36 patients (48.6%), stable disease (SD) in 17 patients (23.0%), and progressive disease (PD) in 11 patients (14.9%). The ORR was 62.1% (46/74) and the disease control rate (DCR) was 85.1% (63/74). Waterfall analysis showed tumor size reduction in 91.9% (57/62) of the patients as per mRECIST (Fig. 2). According to the RECIST v1.1 criteria, the ORR was 16.2% (12/74) and the DCR was 85.1% (63/74) (Table S3). Regardless of whether it was based on mRECIST or RECIST v1.1 criteria, subgroup analyses showed no statistically significant difference in ORR (*P* = 0.732, *P* = 0. 496, respectively) and DCR (*P* = 0.910 for both).

**Table 2:** Best tumor responses as per mRECIST

Best response, *n* (%)	Total (*n* = 74)	LPT (*n* = 38)	LPH (*n* = 12)	LPTH (*n* = 24)	*P* value
CR	10 (13.5)	6 (15.8)	1 (8.3)	3 (12.5)	0.843
PR	36 (48.6)	16 (42.1)	5 (41.7)	15 (62.5)	0.637
SD	17 (23.0)	8 (21.1)	5 (41.7)	4 (16.7)	0.428
PD	11 (14.9)	8 (21.1)	1 (8.3)	2 (8.3)	0.414
ORR	46 (62.1)	22 (57.9)	6 (50.0)	18 (75.0)	0.732
DCR	63 (85.1)	30 (78.9)	11 (91.7)	22 (91.7)	0.910

*Abbreviations*: *CR* complete response, *PR* partial response, *SD* stable disease, *PD* progression disease, *ORR* objective response rate, *DCR* disease control rate, *ORR* CR + PR, *DCR* CR + PR + SD

**Fig. 3:**
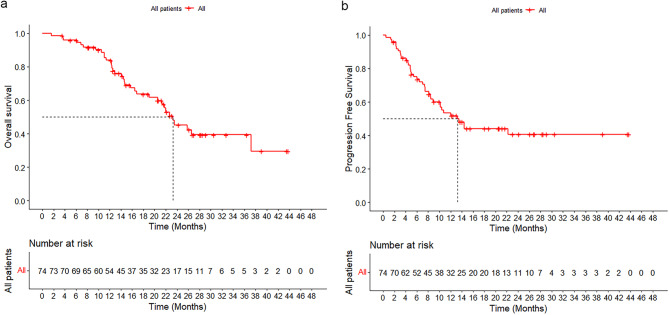
Kaplan-Meier estimates of overall survival (**a**) and progression-free survival (**b**)

**Fig. 4:**
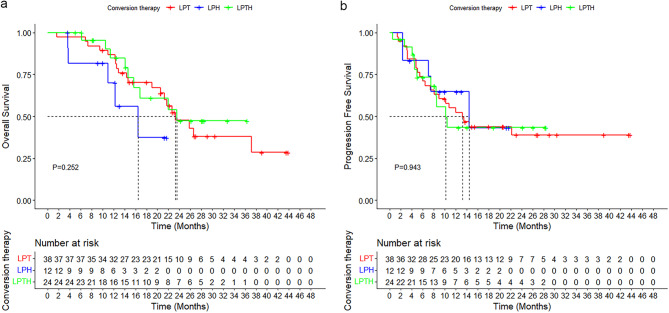
**a** Overall survival and (**b**) progression-free survival of uHCC patients with PVTT who received LPT, LPH, and LPTH regimens

The Median time to response was 2.2 months (IQR, 1.1–10.3 months). As of data cutoff, 38 patients (51.4%) had experienced disease progression and 34 patients (45.9%) had died. The median overall survival (OS) was 23.3 months (95% CI, 18.9–27.7 months), and the median progression-free survival (PFS) was 13.2 months (95% CI, 8.8–17.6 months). The 1-year and 2-year OS rates were 84.0% and 45.3%, respectively, while the 1-year and 2-year PFS rates were 51.8% and 40.7%, respectively (Fig. 3a, b). In the subgroup analysis, the median OS and PFS were 23.3 months (95% CI, 17.7–28.9 months) and 13.2 months (95% CI, 8.0–18.3 months) for the LPT group, 16.5 months (95% CI, 8.1–24.8 months) and14.4 months (95% CI, 1.8–27.0 months) for the LPH group, and 23.5 months (95% CI, not yet determined) and 10.2 months (95% CI, 6.7–13.7 months) for the LPTH group. There were no significant differences in the OS and PFS rates among the LPT, LPH, and LPTH groups (OS: 1 year, 86.8% vs. 70.1% vs. 84.8%; 2 years, 47.6% vs. 37.4% vs. 47.4%, *p* = 0.252; PFS: 1 year, 52.3% vs. 64.8% vs. 43.4%; 2 years, 39.0% vs. 43.2% vs. 43.4%, *p* = 0.943) (Fig. 4a, b).

### Safety

Treatment-related adverse events (TRAEs) after treatment with LPLRT therapy are listed in Table 3. All the 74 (100%) patients reported TRAEs of any grade and 31 (41.9%) patients experienced grade 3–4 TRAEs. The most frequent TRAEs of any grade were increased alanine aminotransferase (71.6%), increased aspartate aminotransferase (68.9%), decreased appetite (58.1%), lymphopenia (56.8%), hypoalbuminemia (55.4%), electrolyte disorder (48.6%), anemia (47.3%), thrombocytopenia (41.9%), fatigue (39.2%), pyrexia (36.5%), leukopenia (33.8%), and nausea (33.8%). The incidence of TRAEs of any grade was 100% in the LPT, LPH, and LPTH subgroups. The incidences of grade 3–4 TRAEs were 44.7% (17/38), 41.7% (5/12), and 37.5% (9/24), respectively (Table S4−6). Six patients had to discontinue or interrupt treatment due to grade 4 TRAEs, which included gastrointestinal bleeding (*n* = 2), autoimmune hepatitis (*n* = 2), anaphylaxis (*n* = 1), and bile leakage (*n* = 1). No grade 5 TRAEs were observed, and all TRAEs were manageable with appropriate treatment.

**Table 3:** Treatment-related adverse events occurring in > 10% of patients

Adverse event	Patients (*n* = 74)				
	Any grade, *n* (%)	Grade 1–2, *n* (%)		Grade 3–4, *n* (%)	
Any adverse event	74 (100.0%)	43 (58.1%)		31 (41.9%)	
Elevated ALT	53 (71.6%)	39 (52.7%)		14 (18.9%)	
Elevated AST	51 (68.9%)	36 (48.6%)		15 (20.3%)	
Decreased appetite	43 (58.1%)	43 (58.1%)		0 (0.0%)	
Lymphopenia	42 (56.8%)	36 (56.8%)		6 (8.1%)	
Hypoalbuminemia	41 (55.4%)	41 (55.4%)		0 (0.0%)	
Electrolytes disorder	36 (48.6%)	33 (44.6%)		3 (4.1%)	
Anaemia	35 (47.3%)	31 (41.9%)		4 (5.4%)	
Thrombocytopenia	31 (41.9%)	28 (37.8%)		3 (4.1%)	
Fatigue	29 (39.2%)	29 (39.2%)		0 (0.0%)	
Pyrexia	27 (36.5%)	22 (29.7%)		5 (6.8%)	
Leukopenia	25 (33.8%)	25 (33.8%)		0 (0.0%)	
Nausea	25 (33.8%)	25 (33.8%)		0 (0.0%)	
Abdominal pain	22 (29.7%)	22 (29.7%)		0 (0.0%)	
Vomiting	22 (29.7%)	22 (29.7%)		0 (0.0%)	
Elevated ALP	21 (28.4%)	21 (28.4%)		0 (0.0%)	
Hypothyroidism	21 (28.4%)	20 (27.0%)		1 (1.4%)	
Hyperbilirubinemia	20 (27.0%)	18 (24.3%)		2 (2.7%)	
Elevated PT	17 (23.0%)	17 (23.0%)		0 (0.0%)	
Hypertension	15 (20.3%)	13 (17.6%)		2 (2.7%)	
Proteinuria	12 (16.2%)	11 (14.9%)		1 (1.4%)	
Weight decreased	10 (13.5%)	10 (13.5%)		0 (0%)	
Rash	9 (12.2%)	9 (12.2%)		0 (0%)	
Diarrhea	9 (12.2%)	8 (10.8%)		1 (1.4%)	
Gum bleeding	8 (10.8%)	8 (10.8%)		0 (0%)	
HFS	8 (10.8%)	6 (8.1%)		2 (2.7%))	

*Abbreviations*: *AST* aspartate aminotransferase, *ALT* alanine aminotransferase, *ALP* alkaline phosphatase, *PT* prothrombin time, *HFS* Hand-foot syndrome

### Convert resection after LPLRT therapy

A total of 31 patients met the resectability criteria, of whom 29 underwent salvage surgery, while 2 continued to receive LPLRT therapy due to refusing surgery. The median interval from initiation of the LPLRT therapy to resection was 3.2 months (IQR, 1.8–12.3 months). The median operation time was 235 min (IQR, 130–356 min), and the median intraoperative blood loss was 300 mL (IQR, 50–2500 mL). Seven patients (24.1%) required intraoperative blood transfusions. Twenty-four patients (82.8%) underwent open hepatectomy, and 26 patients (89.7%) underwent anatomical hepatectomy. All patients achieved R0 resection. Pathological complete remission (pCR) and major pathological response (MPR) were achieved in 10 patients (13.5%) and 9 patients (12.2%), respectively. The remaining 10 patients exhibited varying degrees of pathological remission. Six patients (20.7%) developed Clavien-Dindo IIIa complications, including three cases of ascites with abdominal infection and three cases of pleural effusion with dyspnea. No Clavien-Dindo IIIb-V complications were observed (Table S7). The median postoperative hospital stay was 11 days (IQR, 7–46 days).

Of the patients who underwent surgical resection, 28 (96.6%) received adjuvant therapy. Seventeen patients (58.6%) continued to receive LEN and PD-1, with two of these patients also undergoing TACE once. The remaining 11 patients (37.9%) received either LEN or PD-1 as adjuvant therapy. The mean duration of treatment with LEN after surgery was 6.3 months (IQR, 3.2–11.6 months), and the median number of PD-1 cycles after surgery was 6 (IQR, 3–12 cycles). With a median postoperative follow-up of 19.3 months (IQR, 5.5–39.4 months), seven patients experienced tumor recurrence. Of these, four cases were intrahepatic, one case was extrahepatic, and two cases involved both intrahepatic and extrahepatic recurrence (Table S7). Following recurrence, five patients received LT in addition to the original regimen, including three cases of TACE, one case of radiofrequency ablation, and one case of radiotherapy. In two patients, LEN was switched to regorafenib. Median OS and RFS after surgery were not reached. There were significant differences in the OS and PFS rates between the resection and non-resection groups (OS: 1 year, 96.2% vs. 73.0%; 2 years, 87.0% vs. 17.2%, *p* < 0.001; PFS: 1 year, 81.9% vs. 29.8%; 2 years, 73.7% vs. 10.7%, *p* < 0.001) (Fig, 5a, b).

**Fig. 5:**
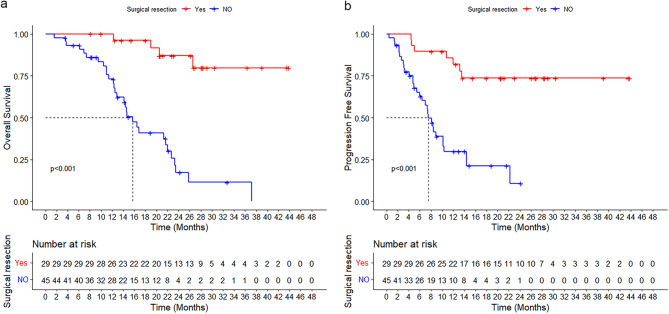
**a** Overall survival and (**b**) progression-free survival of uHCC patients with PVTT who underwent surgical resection versus those who did not

### Risk factor analysis for OS and PFS in all patients

In multivariate analysis, surgical resection (hazard ratio [HR], 0.194; 95% confidence interval [CI], 0.057–0.658; *P* = 0.009), a favorable tumor response according to mRECIST criteria (HR, 0.364; 95% CI, 0.153–0.869; *P* = 0.023), and low HBV-DNA copy number (HR, 0.334; 95% CI, 0.151–0.738; *P* = 0.007) were significantly associated with improved OS (Table 4). Additionally, surgical resection (HR, 0.336; 95% CI, 0.117–0.968; *P* = 0.043), good tumor response according to mRECIST criteria (HR, 0.391; 95% CI, 0.170–0.897; *P* = 0.027), low HBV-DNA copy number (HR, 0.003; 95% CI, 0.178–0.785; *P* = 0.009), and better ECOG-PS (HR, 0.406; 95% CI, 0.178–0.925; *P* = 0.032) were significantly associated with improved PFS (Table S8).

**Table 4:** Univariate and multivariate analysis for overall survival

Characteristics	Univariate analysis	Multivariate analysis		
	*P*‑value	HR	95% CI	*P*‑value
Gender (male vs female)	0.280			
Age (＜60 vs ≥60 years)	0.460			
ECOG PS (0 vs 1)	0.026	0.478	0.211-1.082	0.077
Hepatitis B infection (no vs yes)	0.517			
HBV-DNA copy (<1000 vs ≥1000 copy/mL)	**0.004**	**0.334**	**0.151-0.738**	**0.007**
Comorbidity (no vs yes)	0.467			
Child-Pugh class (A vs B)	0.107			
Baseline AFP (<400 vs ≥400 ng/mL)	0.947			
Baseline PIVKA-II (<400 vs ≥ 400 mAU/mL)	0.295			
Tumor number (Solitary vs Multiple)	0.365			
Maximum tumor size (<10 vs ≥10cm)	0.800			
EHM (no vs yes)	0.630			
PVTT^a^ (Vp2 vs Vp3 vs Vp4)	0.252			
HVTT ^a^ (Vv0-1 vs Vv2 vs Vv3)	0.816			
Treatment regimens (LPT vs LPH vs LPTH)	0.253			
Objective response per RECISTv1.1 criteria (response vs non-response)	0.100			
Objective response per mRECIST criteria (response vs non-response)	**<0.001**	**0.364**	**0.153-0.869**	**0.023**
Surgical resection (yes vs no)	**<0.001**	**0.194**	**0.057-0.658**	**0.009**

*Abbreviations*: *ECOG-PS* Eastern Cooperative Oncology Group performance status, *AFP* α-fetoprotein, *PIVKA-II*, protein induced by vitamin K absence-II, *EHM* Extrahepatic metastasis, *PVTT* Portal vein tumor thrombosis, *HVTT* Hepatic vein tumor thrombosis

^a^Portal vein invasion and Hepatic vein tumor thrombus are graded according to the Liver Cancer Study Group of Japan

## Discussion

This multicenter real-world study provides important insights into the efficacy and safety of the combination of LEN, PD-1, and LRT in patients with initially uHCC with PVTT. The study results demonstrated significant tumor responses, with an ORR of 62.1% and a DCR of 85.1%. Only six patients experienced grade 4 TRAEs, and none encountered the more severe grade 5 TRAEs. All TRAEs were manageable with appropriate treatment. Nearly 40% of patients were able to undergo salvage hepatectomy, with 13.5% achieving a pCR and 12.2% having a confirmed MPR. The median OS was 23.3 months, and the median PFS was 13.2 months. These findings suggest that LPLRT therapy presents a promising treatment option for this difficult-to-treat population. Furthermore, to the best of our knowledge, this study is the first multicenter real-world study to investigate the use of LEN combined with PD-1 and LRT (TACE, HAIC, or TACE-HAIC) as a conversion therapy for uHCC with PVTT.

HCC patients with PVTT often face a severe prognosis and a high risk of death, significantly impairing their quality of life and posing a major health threat [6]. Researchers have been exploring diverse treatment approaches to improve patient survival. Literature reports show significant differences in median OS with various treatments: best supportive care offers a median OS of only 2 to 4 months [27, 28]; sorafenib extends median OS to 10.7 months [29]; TACE results in a median OS of 7 to 10 months [10, 30]; HAIC achieves a median OS of 6.5 to 14 months [31, 32]; and radiation therapy provides a median OS of 9.6 to 10.9 months [32, 33]. Additionally, microwave ablation combined with TACE and TACE combined with sorafenib yield median OS of 13.5 months and 14 months, respectively [34, 35]. Notably, radiofrequency ablation combined with TACE and sorafenib extends median OS to 15.3 months [36], highlighting the potential of combined treatments. Despite these advancements, clinical needs remain unmet, necessitating more effective options. This study introduces an innovative combination treatment strategy for these refractory patients, significantly extending median OS to 23.3 months. This breakthrough not only enhances treatment efficacy but also offers new hope for improving the prognosis of HCC patients with PVTT. Our findings are similar to recent studies on the combination of LEN with PD-1 and TACE or HAIC, which reported median OS of 19.3 and 25 months, respectively [23]. These results demonstrate that LPLRT therapy is a potent treatment for patients uHCC with PVTT, highlighting its significant clinical value and promising application prospects.

Currently, surgery remains the primary treatment to achieve radical cure in patients with HCC [19, 37]. Our study found that nearly 40% of patients with initially uHCC with PVTT were successfully converted to rHCC through LPLRT therapy. This achievement not only broadens the treatment boundaries but also opens up a new potential pathway to radical surgery for these patients. Notably, in the group of patients who underwent salvage surgery after conversion therapy, we observed significantly prolonged OS. Multivariate analysis further confirmed that salvage surgery is an independent favorable factor for improved OS.

However, it is important to recognize that the results of this study alone may not be sufficient to conclusively support the role of salvage surgery in prolonging survival after tumor downstaging through LPLRT conversion therapy. The excellent postoperative survival observed in this study may be partly attributed to the tumor response achieved by the preoperative comprehensive treatment, as most patients who underwent resection had already demonstrated treatment sensitivity (62.1% according to mRECIST). Additionally, pCR was observed in 10 of the 29 patients who underwent hepatectomy, indicating that no viable tumor cells were found in the surgical specimens and suggesting that uHCC with PVTT may potentially be cured by LPLRT alone. Postoperative pathology offers a more accurate assessment of tumor response and better guides subsequent adjuvant treatment [14]. Furthermore, perioperative complications were manageable in patients who successfully achieved conversion and met the criteria for surgical resection. Therefore, we advocate that patients meeting the criteria for surgical resection after conversion therapy should undergo radical surgery whenever possible. Nonetheless, further in-depth clinical studies are required to validate and optimize this treatment strategy.

Previous studies have conclusively shown that the LPH regimen offers significant superiority in OS, PFS, and ORR compared to the LPT regimen in treating patients with advanced HCC with PVTT, underscoring the potential of LPH as a superior treatment option [23]. However, this positive trend was not consistently verified in our detailed subgroup analysis. Specifically, we observed that the LPH and LPTH groups did not demonstrate superiority over the LPT group in terms of both OS and PFS. Similarly, when evaluating ORR to the mRECIST criteria, the LPH group did not demonstrate an improvement over the LPT group, while the LPTH group performed better than both the LPH and LPT groups, though the differences were not statistically significant. We speculate that this may be due to the greater number of tumors in the LPH and LPTH groups compared to the LPT group, which could mask the potential efficacy of the LPH and LPTH regimens in specific treatment settings, leading to the lack of expected improvements in survival and response rates. Additionally, the LPTH group did not show superiority over the LPH group in terms of OS, and PFS, possibly due to the small sample size in our subgroup.

Although this study provides valuable insights into the real-world application of combination therapy for uHCC with PVTT, several limitations need to be addressed. First, the retrospective design may introduce selection bias, limiting the broad applicability of the study’s conclusions. Second, despite the multicenter data collection strategy, the sample size remains insufficient, potentially affecting the statistical power and robustness of the results. Furthermore, the heterogeneity in conversion regimens and various locoregional and systemic treatments may impact the generalizability of the findings. Additionally, the high proportion of HBV-related HCC cases in this study poses challenges in directly applying the results to HCC caused by other etiologies. Finally, the absence of a control group receiving standard treatment limits the ability to directly evaluate the superiority of combination therapy over traditional regimens. However, it is important to note that the multicenter design of this study enhances the generalizability of the results across diverse patient populations. Through in-depth comparative analysis of different subgroups, this study provides detailed data that support the optimization of personalized combination therapy strategies. This is of great significance for guiding clinical practice and promoting advancements in the treatment of HCC.

In conclusion, this multicenter real-world study demonstrated that LPLRT therapy offers a promising treatment option for patients with uHCC with PVTT. The combination therapy exhibited a high tumor response rate, conversion rate, and prolonged survival with manageable safety profiles. Further subgroup analysis indicated that the three different treatment strategies, LPT, LPH, and LPTH, showed comparable efficacy. Notably, salvage surgery is safe and feasible for patients who remain suitable for surgery after LPLRT. Preliminary follow-up data suggest that this approach may improve long-term cancer-free survival, reinforcing its potential value in clinical practice.

## Supplementary Information

Supplementary Material 1.

Supplementary Material 2.

Supplementary Material 3.

Supplementary Material 4.

Supplementary Material 5.

Supplementary Material 6.

Supplementary Material 7.

Supplementary Material 8.

## Data Availability

The datasets generated and/or analyzed during the current study are not publicly available due patients’ individual privacy could be compromised, but are available from the corresponding author on reasonable request.

## References

[1] Sung H, Ferlay J, Siegel RL, Laversanne M, Soerjomataram I, Jemal A, et al. Global cancer statistics 2020: GLOBOCAN estimates of incidence and mortality worldwide for 36 cancers in 185 countries. CA Cancer J Clin. 2021;71(3):209–49. 33538338 10.3322/caac.21660

[2] Villanueva A. Hepatocellular carcinoma. N Engl J Med. 2019;380(15):1450–62. 30970190 10.1056/NEJMra1713263

[3] EASL Clinical Practice Guidelines. Management of hepatocellular carcinoma. J Hepatol. 2018;69(1):182–236. 29628281 10.1016/j.jhep.2018.03.019

[4] Wei X, Jiang Y, Zhang X, Feng S, Zhou B, Ye X, et al. Neoadjuvant three-dimensional conformal radiotherapy for resectable hepatocellular carcinoma with portal vein tumor thrombus: a randomized, open-label, multicenter controlled study. J Clin Oncol. 2019;37(24):2141–51. 31283409 10.1200/JCO.18.02184 PMC6698917

[5] Zhang XP, Gao YZ, Chen ZH, Chen MS, Li LQ, Wen TF, et al. An Eastern hepatobiliary surgery hospital/portal vein tumor thrombus scoring system as an aid to decision making on hepatectomy for hepatocellular carcinoma patients with portal vein tumor thrombus: a multicenter study. Hepatology. 2019;69(5):2076–90. 30586158 10.1002/hep.30490

[6] Xiang X, Lau WY, Wu ZY, Zhao C, Ma YL, Xiang BD, et al. Transarterial chemoembolization versus best supportive care for patients with hepatocellular carcinoma with portal vein tumor thrombus: a multicenter study. Eur J Surg Oncol. 2019;45(8):1460–7. 31005471 10.1016/j.ejso.2019.03.042

[7] Gorodetski B, Chapiro J, Schernthaner R, Duran R, Lin M, Lee H, et al. Advanced-stage hepatocellular carcinoma with portal vein thrombosis: conventional versus drug-eluting beads transcatheter arterial chemoembolization. Eur Radiol. 2017;27(2):526–35. 27277261 10.1007/s00330-016-4445-9 PMC5470590

[8] Venerito M, Pech M, Canbay A, Donghia R, Guerra V, Chatellier G, et al. NEMESIS: noninferiority, individual-patient metaanalysis of selective internal radiation therapy with (90)Y resin microspheres versus Sorafenib in advanced hepatocellular carcinoma. J Nucl Med. 2020;61(12):1736–42. 32358087 10.2967/jnumed.120.242933

[9] Han S, Lee HW, Park JY, Kim SU, Kim DY, Ahn SH, et al. Appraisal of Long-Term outcomes of Liver-Directed concurrent chemoradiotherapy for hepatocellular carcinoma with major portal vein invasion. J Hepatocellular Carcinoma. 2020;7:403–12. 33365287 10.2147/JHC.S276528 PMC7751588

[10] Luo J, Guo RP, Lai EC, Zhang YJ, Lau WY, Chen MS, et al. Transarterial chemoembolization for unresectable hepatocellular carcinoma with portal vein tumor thrombosis: a prospective comparative study. Ann Surg Oncol. 2011;18(2):413–20. 20839057 10.1245/s10434-010-1321-8

[11] Chiang CL, Chiu KWH, Chan KSK, Lee FAS, Li JCB, Wan CWS, et al. Sequential transarterial chemoembolisation and stereotactic body radiotherapy followed by immunotherapy as conversion therapy for patients with locally advanced, unresectable hepatocellular carcinoma (START-FIT): a single-arm, phase 2 trial. Lancet Gastroenterol Hepatol. 2023;8(2):169–78. 36529152 10.1016/S2468-1253(22)00339-9

[12] Yuan Y, He W, Yang Z, Qiu J, Huang Z, Shi Y, et al. TACE-HAIC combined with targeted therapy and immunotherapy versus TACE alone for hepatocellular carcinoma with portal vein tumour thrombus: a propensity score matching study. Int J Surg. 2023;109(5):1222–30. 37026861 10.1097/JS9.0000000000000256 PMC10389515

[13] Qu WF, Ding ZB, Qu XD, Tang Z, Zhu GQ, Fu XT, et al. Conversion therapy for initially unresectable hepatocellular carcinoma using a combination of toripalimab, lenvatinib plus TACE: real-world study. BJS Open. 2022. 10.1093/bjsopen/zrac114. 36125345 10.1093/bjsopen/zrac114 PMC9499852

[14] Wu XK, Yang LF, Chen YF, Chen ZW, Lu H, Shen XY, et al. Transcatheter arterial chemoembolisation combined with lenvatinib plus camrelizumab as conversion therapy for unresectable hepatocellular carcinoma: a single-arm, multicentre, prospective study. EClinMed. 2024;67:102367. 10.1016/j.eclinm.2023.102367 PMC10758712 38169778

[15] Wu JY, Yin ZY, Bai YN, Chen YF, Zhou SQ, Wang SJ, et al. Lenvatinib combined with Anti-PD-1 antibodies plus transcatheter arterial chemoembolization for unresectable hepatocellular carcinoma: A multicenter retrospective study. J Hepatocellular Carcinoma. 2021;8:1233–40. 34676181 10.2147/JHC.S332420 PMC8502053

[16] Wu JY, Zhang ZB, Zhou JY, Ke JP, Bai YN, Chen YF, et al. Outcomes of salvage surgery for initially unresectable hepatocellular carcinoma converted by transcatheter arterial chemoembolization combined with lenvatinib plus anti-PD-1 antibodies: a multicenter retrospective study. Liver Cancer. 2023;12(3):229–37. 37767067 10.1159/000528356 PMC10521320

[17] Ding X, Sun W, Li W, Shen Y, Guo X, Teng Y, et al. Transarterial chemoembolization plus lenvatinib versus transarterial chemoembolization plus sorafenib as first-line treatment for hepatocellular carcinoma with portal vein tumor thrombus: a prospective randomized study. Cancer. 2021;127(20):3782–93. 34237154 10.1002/cncr.33677

[18] Liu BJ, Gao S, Zhu X, Guo JH, Kou FX, Liu SX, et al. Real-world study of hepatic artery infusion chemotherapy combined with anti-PD-1 immunotherapy and tyrosine kinase inhibitors for advanced hepatocellular carcinoma. Immunotherapy. 2021;13(17):1395–405. 34607482 10.2217/imt-2021-0192

[19] Reig M, Forner A, Rimola J, Ferrer-Fàbrega J, Burrel M, Garcia-Criado Á, et al. BCLC strategy for prognosis prediction and treatment recommendation: the 2022 update. J Hepatol. 2022;76(3):681–93. 34801630 10.1016/j.jhep.2021.11.018 PMC8866082

[20] Kudo M, Finn RS, Qin S, Han KH, Ikeda K, Piscaglia F, et al. Lenvatinib versus sorafenib in first-line treatment of patients with unresectable hepatocellular carcinoma: a randomised phase 3 non-inferiority trial. Lancet. 2018;391(10126):1163–73. 29433850 10.1016/S0140-6736(18)30207-1

[21] Finn RS, Qin S, Ikeda M, Galle PR, Ducreux M, Kim TY, et al. Atezolizumab plus bevacizumab in unresectable hepatocellular carcinoma. N Engl J Med. 2020;382(20):1894–905. 32402160 10.1056/NEJMoa1915745

[22] Sun J, Guo R, Bi X, Wu M, Tang Z, Lau WY, et al. Guidelines for diagnosis and treatment of hepatocellular carcinoma with portal vein tumor thrombus in China (2021 edition). Liver Cancer. 2022;11(4):315–28. 35978596 10.1159/000523997 PMC9294940

[23] Lin Z, Chen D, Hu X, Huang D, Chen Y, Zhang J, et al. Clinical efficacy of HAIC (FOLFOX) combined with lenvatinib plus PD-1 inhibitors vs. TACE combined with lenvatinib plus PD-1 inhibitors in the treatment of advanced hepatocellular carcinoma with portal vein tumor thrombus and Arterioportal fistulas. Am J Cancer Res. 2023;13(11):5455–65. 38058801 PMC10695793

[24] Lai Z, He M, Bu X, Xu Y, Huang Y, Wen D, et al. Lenvatinib, toripalimab plus hepatic arterial infusion chemotherapy in patients with high-risk advanced hepatocellular carcinoma: a biomolecular exploratory, phase II trial. Eur J Cancer. 2022;174:68–77. 35981413 10.1016/j.ejca.2022.07.005

[25] Wang L, Feng JK, Lu CD, Wu JY, Zhou B, Wang K, et al. Salvage surgery for initially unresectable HCC with PVTT converted by locoregional treatment plus tyrosine kinase inhibitor and anti-PD-1 antibody. Oncologist. 2024. 10.1093/oncolo/oyae032. 38478404 10.1093/oncolo/oyae032 PMC11299929

[26] Kudo M, Izumi N, Kokudo N, Matsui O, Sakamoto M, Nakashima O, et al. Management of hepatocellular carcinoma in Japan: Consensus-based clinical practice guidelines proposed by the Japan society of hepatology (JSH) 2010 updated version. Dig Dis. 2011;29(3):339–64. 21829027 10.1159/000327577

[27] He C, Ge N, Wang X, Li H, Chen S, Yang Y. Conversion therapy of large unresectable hepatocellular carcinoma with ipsilateral portal vein tumor thrombus using portal vein embolization plus transcatheter arterial chemoembolization. Front Oncol. 2022;12: 923566. 35814420 10.3389/fonc.2022.923566 PMC9261438

[28] Chan SL, Chong CC, Chan AW, Poon DM, Chok KS. Management of hepatocellular carcinoma with portal vein tumor thrombosis: review and update at 2016. World J Gastroenterol. 2016;22(32):7289–300. 27621575 10.3748/wjg.v22.i32.7289 PMC4997643

[29] Llovet JM, Ricci S, Mazzaferro V, Hilgard P, Gane E, Blanc JF, et al. Sorafenib in advanced hepatocellular carcinoma. N Engl J Med. 2008;359(4):378–90. 18650514 10.1056/NEJMoa0708857

[30] Chung GE, Lee JH, Kim HY, Hwang SY, Kim JS, Chung JW, et al. Transarterial chemoembolization can be safely performed in patients with hepatocellular carcinoma invading the main portal vein and may improve the overall survival. Radiology. 2011;258(2):627–34. 21273524 10.1148/radiol.10101058

[31] Eun JR, Lee HJ, Moon HJ, Kim TN, Kim JW, Chang JC. Hepatic arterial infusion chemotherapy using high-dose 5-fluorouracil and cisplatin with or without interferon-alpha for the treatment of advanced hepatocellular carcinoma with portal vein tumor thrombosis. Scand J Gastroenterol. 2009;44(12):1477–86. 19958061 10.3109/00365520903367262

[32] Hamaoka M, Kobayashi T, Kuroda S, Iwako H, Okimoto S, Kimura T, et al. Hepatectomy after down-staging of hepatocellular carcinoma with portal vein tumor thrombus using chemoradiotherapy: a retrospective cohort study. Int J Surg. 2017;44:223–8. 28676383 10.1016/j.ijsu.2017.06.082

[33] Nakazawa T, Hidaka H, Shibuya A, Okuwaki Y, Tanaka Y, Takada J, et al. Overall survival in response to Sorafenib versus radiotherapy in unresectable hepatocellular carcinoma with major portal vein tumor thrombosis: propensity score analysis. BMC Gastroenterol. 2014;14:84. 24886354 10.1186/1471-230X-14-84 PMC4014748

[34] Zhu K, Chen J, Lai L, Meng X, Zhou B, Huang W, et al. Hepatocellular carcinoma with portal vein tumor thrombus: treatment with transarterial chemoembolization combined with sorafenib–a retrospective controlled study. Radiology. 2014;272(1):284–93. 24708192 10.1148/radiol.14131946

[35] Long J, Zheng JS, Sun B, Lu N. Microwave ablation of hepatocellular carcinoma with portal vein tumor thrombosis after transarterial chemoembolization: a prospective study. Hepatol Int. 2016;10(1):175–84. 26742761 10.1007/s12072-015-9673-6

[36] Ding X, Sun W, Chen J, Li W, Shen Y, Guo X, et al. Percutaneous radiofrequency ablation combined with transarterial chemoembolization plus Sorafenib for large hepatocellular carcinoma invading the portal venous system: a prospective randomized study. Front Oncol. 2020;10: 578633. 33194699 10.3389/fonc.2020.578633 PMC7644860

[37] Zhou J, Sun H, Wang Z, Cong W, Wang J, Zeng M, et al. Guidelines for the diagnosis and treatment of hepatocellular carcinoma (2019 edition). Liver Cancer. 2020;9(6):682–720. 33442540 10.1159/000509424 PMC7768108

