# Supplementary material for: Lenvatinib combined with anti-PD-1 antibodies plus locoregional treatment for initial unresectable hepatocellular carcinoma with portal vein tumor thrombosis: a multicenter real-world study
Source: BMC Cancer. 2025 Jul 10;25:1162. doi: 10.1186/s12885-025-14543-9 (PMC12247254; doi:10.1186/s12885-025-14543-9)
Supplement: Supplementary file 1 — Supplementary Material 1. [file 12885_2025_14543_MOESM1_ESM.docx]

Table S1

Comparison of the number of tumors between subgroups

| Characteristic | LPT (n=38) | LPH (n=12) | *P* value |
| --- | --- | --- | --- |
| Tumor number, n (%) |  |  | 0.047 |
| Solitary | 22 | 3 |  |
| Multiple | 16 | 9 |  |

| Characteristic | LPT (n=38) | LPTH (n=24) | *P* value |
| --- | --- | --- | --- |
| Tumor number, n (%) |  |  | 0.027 |
| Solitary | 22 | 7 |  |
| Multiple | 16 | 17 |  |

| Characteristic | LPH (n=12) | LPTH (n=24) | *P* value |
| --- | --- | --- | --- |
| Tumor number, n (%) |  |  | 1.000* |
| Solitary | 3 | 7 |  |
| Multiple | 9 | 17 |  |

Note: * Fisher's exact test.
