# Supplementary material for: Lenvatinib combined with anti-PD-1 antibodies plus locoregional treatment for initial unresectable hepatocellular carcinoma with portal vein tumor thrombosis: a multicenter real-world study
Source: BMC Cancer. 2025 Jul 10;25:1162. doi: 10.1186/s12885-025-14543-9 (PMC12247254; doi:10.1186/s12885-025-14543-9)
Supplement: Supplementary file 2 — Supplementary Material 2. [file 12885_2025_14543_MOESM2_ESM.docx]

| Table S2  Summary of treatment regimens. | | | | |  |  |
| --- | --- | --- | --- | --- | --- | --- |
| Regimens | Total  (n=74) | LPT  (n=38) | LPH  (n=12) | LPTH  (n=24) | | *P* value |
| PD-1 class, n (%) |  |  |  |  | |  |
| Camrelizumab | 48 (64.9) | 26 (68.4) | 9 (75.0) | 13 (54.2) | | 0.808 |
| Tislelizumab | 16 (21.6) | 10 (26.3) | 2 (16.7) | 4 (16.7) | | 0.720 |
| Sintilimab | 9 (12.2) | 2 (5.3) | 1 (8.3) | 6 (25.0) | | 0.123* |
| Toripalimab | 1 (1.3) | 0 (0.0) | 0 (0.0) | 1 (4.1) | | 0.493* |
| Duration of LEN (months), median (IQR) | 2.2 (1.1-15.7) | 2.2 (1.1-14.2) | 2.6 (1.9-5.6) | 2.3 (1.4-15.7) | | 0.943 |
| Cycles of PD-1s, median (IQR) | 3 (1-22) | 3 (1-20) | 3 (2-7) | 3 (2-22) | | 0.459* |
| Number of TACE, median (IQR) | 2 (1-6) | 2 (1-6) | - | 2 (1-6) | | - |
| Number of HAIC, median (IQR) | 2 (1-6) | - | 3 (1-6) | 2 (1-5) | | - |

Note: * Fisher's exact test.

Abbreviations: LEN, Lenvatinib; PD-1, Anti-PD-1 Antibodies; HAIC, hepatic arterial infusion chemotherapy; TACE, transcatheter arterial chemoembolization;
