# Supplementary material for: Lenvatinib combined with anti-PD-1 antibodies plus locoregional treatment for initial unresectable hepatocellular carcinoma with portal vein tumor thrombosis: a multicenter real-world study
Source: BMC Cancer. 2025 Jul 10;25:1162. doi: 10.1186/s12885-025-14543-9 (PMC12247254; doi:10.1186/s12885-025-14543-9)
Supplement: Supplementary file 3 — Supplementary Material 3. [file 12885_2025_14543_MOESM3_ESM.docx]

| Table S3  Best tumor responses as per RECIST. | | | | | |
| --- | --- | --- | --- | --- | --- |
| Best Response, n (%) | Total (n=74) | LPT (n=38) | LPH (n=12) | LPTH (n=24) | *P* value |
| CR, n (%) | 0 (0.0) | 0 (0.0) | 0 (0.0) | 0 (0.0) | - |
| PR, n (%) | 12 (16.2) | 4 (10.5) | 3 (25.0) | 5 (20.8) | 0.496 |
| SD, n (%) | 51 (68.9) | 26 (68.4) | 8 (66.7) | 17 (70.9) | 0.993 |
| PD, n (%) | 11 (14.9) | 8 (21.1) | 1 (8.3) | 2 (8.3) | 0.414 |
| ORR, n (%) | 12 (16.2) | 4 (10.5) | 3 (25.0) | 5 (20.8) | 0.496 |
| DCR, n (%) | 63 (85.1) | 30 (78.9) | 11 (91.7) | 22 (91.7) | 0.910 |

Abbreviations: CR, complete response; PR, partial response; SD, stable disease; PD, progression disease; ORR, objective response rate; DCR, disease control rate; ORR = C R + PR; DCR = CR + PR + SD.
