# Supplementary material for: Lenvatinib combined with anti-PD-1 antibodies plus locoregional treatment for initial unresectable hepatocellular carcinoma with portal vein tumor thrombosis: a multicenter real-world study
Source: BMC Cancer. 2025 Jul 10;25:1162. doi: 10.1186/s12885-025-14543-9 (PMC12247254; doi:10.1186/s12885-025-14543-9)
Supplement: Supplementary file 4 — Supplementary Material 4. [file 12885_2025_14543_MOESM4_ESM.docx]

| Table S4  Treatment-related adverse events occurring in>10% of patients with LPT regimen. | | | | | |
| --- | --- | --- | --- | --- | --- |
| Adverse Event | Patients (n=38) | |  | |  |
|  | Any grade, n (%) | Grade1–2, n (%) | | Grade3–4, n (%) | |
| Any adverse event | 38 (100.0%) | 21(55.3%) | | 17 (44.7%) | |
| Elevated AST | 26 (68.5%) | 18 (47.4%) | | 8 (21.1%) | |
| Elevated ALT | 24 (63.2%) | 19 (50.0%) | | 5 (13.2%) | |
| Decreased appetite | 21 (55.3%) | 21 (55.3%) | | 0 (0.0%) | |
| Hypoalbuminemia | 18 (47.4%) | 18 (47.4%) | | 0 (0.0%) | |
| Lymphopenia | 18 (47.4%) | 17 (44.7%) | | 1 (2.6%) | |
| Electrolytes disorder | 16 (42.1%) | 15 (39.5%) | | 1 (2.6%) | |
| Anaemia | 16 (42.1%) | 15 (39.5%) | | 1 (2.6%) | |
| Fatigue | 15 (39.5%) | 15 (39.5%) | | 0 (0.0%) | |
| Thrombocytopenia | 15 (39.5%) | 14 (36.8%) | | 1 (2.6%) | |
| Pyrexia | 14 (36.8%) | 13 (34.2%) | | 1 (2.6%) | |
| Nausea | 12 (31.6%) | 12 (31.6%) | | 0 (0.0%) | |
| Leukopenia | 11 (29.0%) | 11 (29.0%) | | 0 (0.0%) | |
| Abdominal pain | 10 (26.3%) | 10 (26.3%) | | 0 (0.0%) | |
| Vomiting | 10 (26.3%) | 10 (26.3%) | | 0 (0.0%) | |
| Elevated ALP | 10 (26.3%) | 10 (26.3%) | | 0 (0.0%) | |
| Hypothyroidism | 10 (26.3%) | 9 (23.7%) | | 1 (2.6%) | |
| Hyperbilirubinemia | 10 (26.3%) | 9 (23.7%) | | 1 (2.6%) | |
| Elevated PT | 9 (23.7%) | 9 (23.7%) | | 0 (0.0%) | |
| Hypertension | 8 (21.1%) | 8 (21.1%) | | 0 (0.0%) | |
| Proteinuria | 6 (15.8%) | 6 (15.8%) | | 0 (1.4%) | |
| Weight decreased | 5 (13.2%) | 5 (13.2%) | | 0 (0.0%) | |
| Rash | 5 (13.2%) | 5 (13.2%) | | 0 (0.0%) | |
| Diarrhea | 5 (13.2%) | 4 (10.5%) | | 1 (2.6%) | |
| Gum bleeding | 4 (10.5%) | 4 (10.5%) | | 0 (0.0%) | |
| HFS | 4 (10.5%) | 3 (7.9%) | | 1 (2.6%) | |

Abbreviations: AST, aspartate aminotran sferase; ALT, alanine aminotransferase; ALP, alkaline phosphatase; PT, prothrombin time; HFS, Hand-foot syndrome.
