# Supplementary material for: Lenvatinib combined with anti-PD-1 antibodies plus locoregional treatment for initial unresectable hepatocellular carcinoma with portal vein tumor thrombosis: a multicenter real-world study
Source: BMC Cancer. 2025 Jul 10;25:1162. doi: 10.1186/s12885-025-14543-9 (PMC12247254; doi:10.1186/s12885-025-14543-9)
Supplement: Supplementary file 6 — Supplementary Material 6. [file 12885_2025_14543_MOESM6_ESM.docx]

| Table S6  Treatment-related adverse events occurring in>10% of patients with LPTH regimen. | | | | | |
| --- | --- | --- | --- | --- | --- |
| Adverse Event | Patients (n=24) | |  | |  |
|  | Any grade, n (%) | Grade1–2, n (%) | | Grade3–4, n (%) | |
| Any adverse event | 24 (100.0%) | 15 (62.5%) | | 9 (37.5%) | |
| Elevated ALT | 20 (83.3%) | 13 (54.2%) | | 7 (29.3%) | |
| Elevated AST | 17 (70.8%) | 13 (54.2%) | | 4 (16.7%) | |
| Lymphopenia | 17 (70.8%) | 13 (54.2%) | | 4 (16.7%) | |
| Decreased appetite | 15 (62.5%) | 15 (62.5%) | | 0 (0.0%) | |
| Hypoalbuminemia | 15 (62.5%) | 15 (62.5%) | | 0 (0.0%) | |
| Electrolytes disorder | 15 (62.5%) | 13 (54.2%) | | 2 (8.3%) | |
| Anaemia | 14 (58.3%) | 11 (45.8%) | | 3 (12.5%) | |
| Leukopenia | 10 (41.7%) | 10 (41.7%) | | 0 (0.0%) | |
| Thrombocytopenia | 10 (41.7%) | 9 (37.5%) | | 1 (4.2%) | |
| Fatigue | 9 (37.5%) | 9 (37.5%) | | 0 (0.0%) | |
| Nausea | 9 (37.5%) | 9 (37.5%) | | 0 (0.0%) | |
| Abdominal pain | 9 (37.5%) | 9 (37.5%) | | 0 (0.0%) | |
| Vomiting | 9 (37.5%) | 9 (37.5%) | | 0 (0.0%) | |
| Pyrexia | 9 (37.5%) | 5 (20.8%) | | 4 (16.7%) | |
| Elevated ALP | 8 (33.3%) | 8 (33.3%) | | 0 (0.0%) | |
| Hypothyroidism | 7 (29.2%) | 7 (29.2%) | | 0 (0.0%) | |
| Elevated PT | 6 (25.0%) | 6 (25.0%) | | 0 (0.0%) | |
| Hyperbilirubinemia | 6 (25.0%) | 5 (20.8%) | | 1 (4.2%) | |
| Hypertension | 5 (20.8%) | 4 (16.7%) | | 1 (4.2%) | |
| Rash | 4 (16.7%) | 4 (16.7%) | | 0 (0.0%) | |
| Diarrhea | 4 (16.7%) | 4 (16.7%) | | 0 (0.0%) | |
| Gum bleeding | 4 (16.7%) | 4 (16.7%) | | 0 (0.0%) | |
| Proteinuria | 4 (16.7%) | 3 (12.5%) | | 1 (4.2%) | |
| HFS | 4 (16.7%) | 3 (12.5%) | | 1 (4.2%) | |
| Weight decreased | 3 (12.5%) | 3 (12.5%) | | 0 (0.0%) | |

Abbreviations: AST, aspartate aminotransferase; ALT, alanine aminotransferase; ALP, alkaline phosphatase; PT, prothrombin time; HFS, Hand-foot syndrome.
