# Supplementary material for: Lenvatinib combined with anti-PD-1 antibodies plus locoregional treatment for initial unresectable hepatocellular carcinoma with portal vein tumor thrombosis: a multicenter real-world study
Source: BMC Cancer. 2025 Jul 10;25:1162. doi: 10.1186/s12885-025-14543-9 (PMC12247254; doi:10.1186/s12885-025-14543-9)
Supplement: Supplementary file 7 — Supplementary Material 7. [file 12885_2025_14543_MOESM7_ESM.docx]

| Table S7  Perioperative outcomes of salvage surgery after conversion therapy. | |  |
| --- | --- | --- |
| Perioperative outcomes, n (%) | Patients (n=29) | |
| Surgical conversion rate, n/N (%) | 29/74 (39.2%) | |
| Time from the start of conversion therapy to surgical resection (months), median (IQR) | 3.2 (1.8, 12.3) | |
| Surgical type, n (%) |  | |
| Laparoscopic | 5 (17.2%) | |
| Open | 24 (82.8%) | |
| Hepatectomy |  | |
| Anatomical | 26 (89.7%) | |
| Non-anatomical | 3 (10.3%) | |
| Operative time (min), median (IQR) | 235 (130, 356) | |
| Blood loss (mL), median (IQR) | 300 (50, 2500) | |
| Intraoperative blood transfusion |  | |
| Yes | 7 (24.1%) | |
| No | 22 (75.9%) | |
| Difficulty of operation, n (%) |  | |
| Increased | 0 (0.0%) | |
| Not increased | 29 (100.0%) | |
| R0 resection rate, n (%) | 29 (100.0%) | |
| pCR, n (%) | 10 (13.5%) | |
| MPR, n (%) | 9 (12.2%) | |
| Clavien-Dindo classification, n (%) |  | |
| 0-II | 23 (79.3%) | |
| III-V | 6 (20.7%) | |
| Postoperative hospital stays (days), median (IQR) | 11 (7 - 46) | |
| Time of postoperative follow-up (months), median (IQR) | 19.3 (5.5, 39.4) | |
| Number of patients received adjuvant therapy, n (%) | 28 (96.6%) | |
| LEN + PD-1 | 15 (51.7%) | |
| LEN + PD-1 + TACE | 2 (6.9%) | |
| LEN | 10 (34.5%) | |
| PD-1 | 1 (3.4%) | |
| Adjuvant therapy |  | |
| Cycles of PD-1, median (IQR) | 6 (3 - 12) | |
| Duration of LEN (months), median (IQR) | 6.3 (3.2,11.6) | |
| Number of patients with HCC recurrence, n (%) | 7 (24.1%) | |
| Intrahepatic | 4 (13.8%) | |
| Pulmonary | 1 (3.4%) | |
| Both | 2 (6.9%) | |

Abbreviations: pCR, pathologic complete response; MPR, major pathologic response; LEN, Lenvatinib; PD-1, Anti-PD-1 Antibodies; TACE, transcatheter arterial chemoembolization;
