# Supplementary material for: Lenvatinib combined with anti-PD-1 antibodies plus locoregional treatment for initial unresectable hepatocellular carcinoma with portal vein tumor thrombosis: a multicenter real-world study
Source: BMC Cancer. 2025 Jul 10;25:1162. doi: 10.1186/s12885-025-14543-9 (PMC12247254; doi:10.1186/s12885-025-14543-9)
Supplement: Supplementary file 8 — Supplementary Material 8. [file 12885_2025_14543_MOESM8_ESM.docx]

| Table S8  Univariate and multivariate analysis for progression free survival. | | | | |  |
| --- | --- | --- | --- | --- | --- |
| Characteristics | Univariate analysis | Multivariate analysis | | | |
|  | *P*‑value | HR | 95% CI | *P*‑value | |
| Gender (male vs female) | 0.277 |  |  |  | |
| Age (＜60 vs ≥60 years) | 0.654 |  |  |  | |
| ECOG PS (0 vs 1) | **0.006** | **0.406** | **0.178-0.925** | **0.032** | |
| Hepatitis B infection (no vs yes) | 0.229 |  |  |  | |
| HBV-DNA copy  (<1000 vs ≥1000 copy/mL) | **0.003** | **0.374** | **0.178-0.785** | **0.009** | |
| Comorbidity (no vs yes) | 0.460 |  |  |  | |
| Child-Pugh class (A vs B) | 0.236 |  |  |  | |
| Baseline AFP (<400 vs ≥400 ng/mL) | 0.763 |  |  |  | |
| Baseline PIVKA-II (<400 vs ≥ 400 mAU/mL) | 0.196 |  |  |  | |
| Tumor number (Solitary vs Multiple) | 0.523 |  |  |  | |
| Maximum tumor size (<10 vs ≥10cm) | 0.988 |  |  |  | |
| EHM (no vs yes) | 0.699 |  |  |  | |
| PVTT ^a^ (Vp2 vs Vp3 vs Vp4) | 0.962 |  |  |  | |
| HVTT ^a^ (Vv0-1 vs Vv2 vs Vv3) | 0.548 |  |  |  | |
| Treatment regimens (LPT vs LPH vs LPTH group) | 0.943 |  |  |  | |
| Objective response per RECISTv1.1 criteria (response vs non-response) | 0.254 |  |  |  | |
| Objective response per mRECIST criteria (response vs non-response) | **<0.001** | **0.391** | **0.170-0.897** | **0.027** | |
| Surgical resection (yes vs no) | **<0.001** | **0.336** | **0.117-0.968** | **0.043** | |

Note: ^a^ Portal vein invasion and Hepatic vein tumor thrombus are graded according to the Liver Cancer Study Group of Japan.

Abbreviations: ECOG-PS, Eastern Cooperative Oncology Group performance status; AFP, α-fetoprotein; PIVKA-II, protein induced by vitamin K absence-II; EHM, Extrahepatic metastasis; PVTT, Portal vein tumor thrombosis; HVTT, Hepatic vein tumor thrombosis;
